# Preventing and Addressing Interpersonal Conflicts Among Residents in Diverse Nursing Home Settings

**DOI:** 10.1093/geroni/igaf122.872

**Published:** 2025-12-31

**Authors:** Amy Roberts, Lirisha Tuladhar, Veronica Barrios, Dongxia Sang, E-Shien Chang

**Affiliations:** Miami University, Oxford, Ohio, United States; Miami University, Oxford, Ohio, United States; Miami University, Oxford, Ohio, United States; Miami University, Oxford, Ohio, United States; Weill Cornell Medicine, New York, New York, United States

## Abstract

This study examines how nursing home staff can prevent and address interpersonal conflicts among residents in long-term care facilities. As these facilities increasingly serve diverse populations, understanding conflict dynamics is crucial for promoting a sense of belonging, acceptance, and respect. Interviews with twelve social services workers (with 1–25 years of experience) from nursing homes across the U.S. revealed that conflicts often stem from cultural misunderstandings and differences in social identities. Participants highlighted the importance of trauma-informed, person-centered care and suggested increased monitoring, early intervention, and environmental adjustments (e.g., changing roommates) as strategies to prevent or resolve resident-to-resident conflicts. Four facilities had formal inclusion programs with staff or resident leaders to help new residents adjust and socially integrate. While many described efforts to incorporate culturally appropriate activities, match roommates based on shared interests and preferences, and provide dementia training for staff, only one respondent reported participating in a program aimed at addressing unconscious biases among staff. Overall, respondents felt that there is limited training available for staff on how to help residents live harmoniously with others who differ from them. Factors contributing to resident conflicts included cultural differences (n = 9), cognitive impairments (n = 9), disruptive behavior (n = 7), race (n = 6), younger age (n = 5), religion (n = 5), political divisions (n = 2), gender identity (n = 1), and substance use (n = 1). The findings suggest a need for staff training on managing high-conflict situations and effectively engaging with residents from diverse backgrounds, using strategies such as active listening, motivational interviewing, and goal-setting.

